# Series of screening compounds with high hit rates for the exploration of multi-target activities and assay interference

**DOI:** 10.4155/fsoa-2017-0137

**Published:** 2018-01-05

**Authors:** Dagmar Stumpfe, Erik Gilberg, Jürgen Bajorath

**Affiliations:** 1 Department of Life Science Informatics, B-IT, LIMES Program Unit Chemical Biology & Medicinal Chemistry, Rheinische Friedrich-Wilhelms-Universität, Dahlmannstr 2, D-53113 Bonn, Germany

**Keywords:** analog series, assay interference, biological screening assays, extensively tested active compounds, hit rate statistics, multi-target activities, open access data

## Abstract

**Aim:**

Generation of a database of analog series (ASs) with high assay hit rates for the exploration of assay interference and multi-target activities of compounds.

**Methodology:**

ASs were computationally extracted from extensively tested screening compounds with high hit rates.

**Data:**

A total of 6941 ASs were assembled comprising 14,646 unique compounds that were tested in a total of 1241 different assays covering 426 specified targets. These ASs were organized and prioritized on the basis of different activity and assay frequency criteria. All ASs and associated information are made available in an open access deposition.

**Next steps:**

The large set of ASs will be further analyzed computationally and from a chemical perspective to identify assay interference compounds and candidates for exploring target promiscuity.

## Lay abstract

In drug discovery, compounds are tested in high-throughput screening assays for desired biological activities. Unfortunately, some test compounds that are not specifically active give false-positive (artificial) activity signals in assays, which are difficult to recognize. However, other compounds can bind specifically to multiple targets and hence give true activity signals in multiple assays. To help distinguish between artifacts and true activities, we have systematically identified series of analogs (i.e., structurally closely related compounds) that are active with unusually high frequency across biological assays. Herein, we describe these data and make them freely available for follow-up analysis.

## Graphical abstract

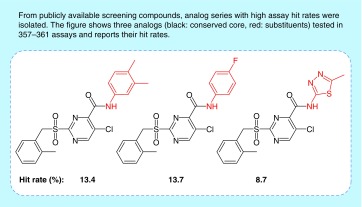

**Key terms**

**Analog series (AS):** Series of closely related compounds sharing the same core structure and having different substituents (R-groups) at one or more sites (substitution sites). In this study, only ASs with a single substitution site were considered.

**Hit rate (HR):** Defined here as the proportion of assays in which a screening compound was active.

**Matching molecular series (MMS):** A series of two or more compounds that are only distinguished by a chemical modification at a single site. The MMS represents a computational data structure for the systematic extraction of ASs with single substitution sites from large compound sets.

Multi-target activities of small molecules experience increasing interest in pharmaceutical research, especially in the context of polypharmacology [1–3]. For example, small subsets of highly promiscuous kinase inhibitors have become a paradigm for polypharmacological drug action in cancer treatment [4]. However, true multi-target activities of compounds must be clearly distinguished from nonspecific interactions and other assay artifacts [5,6], which represent a general problem for biological screening and medicinal chemistry. Compounds that are likely to be reactive or autofluorescent under assay conditions have high potential to cause false-positive activity signals. Such assay interference compounds have been investigated in specific assay formats and instructive case studies, for example [6–8], and have also been explored computationally by compound data mining, for example, [9]. However, the study of assay interference is far from being a trivial task since compounds with interference potential typically occur as substructures in larger molecules and their potential reactivities tend to depend on the structural context in which they are presented [9]. Recently, we have reported a large-scale statistical analysis of publicly available screening data to identify extensively tested analog series (ASs) with high hit rates (HRs) across assays [10]. The major goal of this study was the identification of the most frequently active ASs. Collecting such series was thought to provide a general basis for further exploring assay interference of screening compounds versus multi-target activities. Our analysis identified thousands of ASs with unusually high HRs considering the global HR distribution over all screening assays [10]. In this data note, we report an open access deposition of all qualifying ASs and associated assay frequency and activity data. The ASs were organized and prioritized according to different parameters, as discussed in the following.

## Methodology

In the following, a summary of the methodological framework of our ASs and HR analysis is provided, yielding the collection of most frequently active ASs that is made freely available. For further methodological details, the interested reader is referred to the original publication [10].

### Definition of analog series

To generate ASs, matching molecular series (MMSs) [11] were systematically extracted from selected screening compounds. MMS is an extension of the matched molecular pair formalism [12] and defined as a series of compounds that share the same core structure and are distinguished at a single substitution site [11]. MMSs are algorithmically extracted from compound sources in an efficient manner [11]. For their generation, retrosynthetic rules and size restrictions for substituents were applied [10] such that MMSs represented typically observed ASs with a single substitution site.

So defined ASs with single-site modifications provided HR controls for closely related analogs. For example, if closely related compounds comprising a series are active in a wide variety of assays, interference potential is very likely. However, if they are consistently active against the same set of targets, promiscuity should be further explored. Furthermore, if some analogs are frequently active but others are not, questions concerning experimental data and confidence levels might be raised. Given this important control opportunity provided by comparing analogs, ASs instead of individual compounds were identified to represent the basic structural unit for our study.

### Statistical analysis

From PubChem Bioassays [13], the major public repository for biological screening data, a set of 437,257 compounds was preselected that were extensively tested in primary and confirmatory assays, with a median value of 347 assays per compound. The frequency distribution for the much larger number of primary assays was analyzed and a subset of 327,532 compounds selected that were each tested in more than 257 primary assays, with a median of 426 assays per compound. For this subset, the HR distribution over all primary assays was analyzed, yielding a median HR of 0.4% and a third quartile HR of 1.0%. On the basis of this distribution, 80,495 compounds with HR >1.0% were taken and their HR distribution was separately analyzed, yielding a median HR of 1.8%. From this subset, 41,609 compounds with HR >1.8% were then selected as the subset having highest HRs in primary screening assays, with a median HR of 2.9%. More than 93% of these compounds were also active in confirmatory assays. From these 41,609 screening compounds with overall highest HRs, MMSs were systematically extracted.

### Series parameters

Three parameters were introduced to characterize MMSs/ASs and calculated as follows:

- *Cumulative series hit rate*
  The number of unique assays in which one or more analogs comprising a series were active divided by the number of unique assays in which all analogs were tested;
- *Assay overlap*
  The proportion of assays in which all analogs of a series were tested (shared assays) relative to the union of unique assays;
- *Assays with inconsistent activity*
  The proportion of shared assays in which different analogs of a series were active and inactive.

## Data

### Analog series with high hit rates

From the 41,609 extensively tested compounds with HR >1.8%, a total of 6941 ASs of varying size (two to 17 analogs) were extracted, which contained a total of 17,599 compounds (14,646 unique compounds; 2953 participated in multiple ASs). The 6941 ASs were tested in a total of 1241 assay covering 426 specified targets. Distributions of assay- and target-based HRs for ASs were similar, yielding median values of 5.8% and 4.9%, respectively. Filtering for assay interference candidates [6,14] revealed that 5065 ASs did not contain any known interference compounds. Hence, these ASs should provide a substantial resource for follow-up analysis. ASs statistics and a data summary are given in Table 1.

**Table 1.** **Analog series, compounds, assays and hit rates.**

**AS**	

Total	6941

**Compounds**	

Total	17,599

Unique	14,646

Per AS	2–17

**Assays**	

Total	1213

Per AS	261–592

**Assays with activity**	

Total	1111

Per AS	5–148

**HR**	

Per AS	>1.8%–32.9%

Statistics are provided for AS extracted from screening compounds with overall highest assay HR.

AS: Analog series; HR: Hit rate.

**Figure 1.**
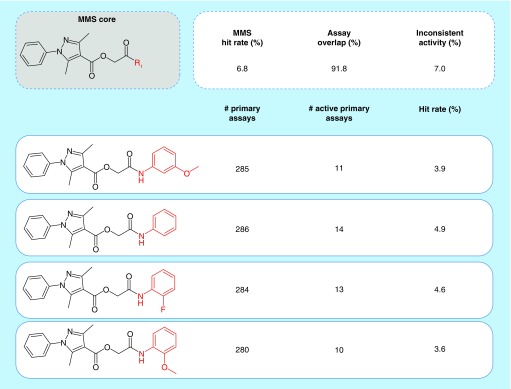
**Exemplary analog series.** Shown is an analog series with high hit rate and high priority for follow-up analysis. The conserved (MMS) core is colored black and substituents are colored red. Assay frequencies and series parameters are provided. MMS: Matching molecular series.

ASs with high priority for follow-up analysis of multi-target activities and/or assay interference should best display high cumulative HR, high assay overlap and low proportion of assays with inconsistent activity. In other words, analogs comprising a prioritized AS should be tested in as many shared assays as possible, be frequently active, and exhibit consistent assay activity profiles. Figure 1 shows an exemplary AS meeting these criteria.

The ASs were separately ranked according to criteria 1–3 described above and a rank fusion was calculated to globally organize ASs. Following rank fusion, series were ordered and prioritized according to the smallest sum of ranks (consensus rank).

### Open access deposition

All 6941 ASs and the associated assay and activity information have been made freely available as a deposition on the ZENODO open access platform [15]. The deposition contains two text files with ASs and supplementary compound and target information:

- ***AS.txt***
  This file contains all qualifying ASs. Each column is separated by a tab. For each AS, the following information is provided:
  - a.1. Individual identifier (see: ‘AS_ID’).
  - a.2. Consensus rank following rank fusion (see: ‘Rank’).
  - a.3. MMS core in aromatic, nonstereo SMILES [16] representation (see: ‘MMS_core’).
  - a.4. Cumulative AS hit rate (see: AS_HR [%]), assay overlap (see: ‘Assay_OV [%]’) and proportion of assays with inconsistent activity (see: ‘Inconsistent_activity [%]’).
  - a.5. Identifier (ID) for each AS compound, comma separated (see: ‘CID’). The CID gives the PubChem compound ID.
  - a.6. Aromatic, nonstereo SMILES representation of each AS compound, comma separated (see: ‘AromaticNon-StereoSMILES’).
- ***AS_SupportingInformation.txt***
  This file contains detailed information about the 17,599 compounds forming the ASs. Each column is separated by a tab. For each compound, the following information is provided:
  - b.1. Individual PubChem compound ID (see: ‘CID’).
  - b.2. Aromatic, nonstereo SMILES representation of the compound (see: ‘AromaticNon-StereoSMILES’).
  - b.3. ID of the AS the compound belongs to (see: ‘AS_ID’).
  - b.4. AS consensus rank following rank fusion (see: ‘Rank’).
  - b.5. MMS core in aromatic, nonstereo SMILES representation (see: ‘MMS_core’).
  - b.6. Number of primary assays in which the compound was tested (see: ‘#primary_assays’).
  - b.7. Number of primary assays in which the compound was active (see: ‘#active_primary_assays’).
  - b.8. The hit rate of the compound across primary assays (see: ‘Hit_rate(%)’).
  - b.9. PubChem GI (ID) numbers for targets the compound was active against, comma separated (see: ‘Target_ID’).
  - b.10. Compound detected by assay interference filters (yes/no)? (see: ‘Interference CPD’).
    We note that the current Zenodo deposition [15] contains two versions 1 and 2. The original version 1 was updated to include b.10 (version 2).

## Summary & next steps

The AS organization of screening compounds with high HRs was intended to provide structural context information for a further evaluation of assay interference potential and multi-target activities. The selection process was exclusively based on HRs and structural relationships, without preconceived notion of assay interference or target promiscuity. Since compounds forming the ASs were selected to have statistically highest hit rates across primary assays, it is anticipated that series might contain more previously unrecognized interference compounds than candidates for polypharmacology. This hypothesis will be evaluated by an in-depth analysis of the organized ASs from a chemical perspective. Making the ASs and associated information publicly available provides an unbiased basis for computational and knowledge-based follow-up analyses in different laboratories. This is expected to enable further progress in rationalizing undesired assay interference characteristics and differentiating them from true multi-target activities of small molecules.

**Executive summary**

- Compounds with multi-target activity provide the basis for polypharmacology.
- Assay artifacts caused by interference compounds represent substantial problems for biological screening and medicinal chemistry.

**Methodology**

- A statistical analysis of assay hit rates (HRs) of extensively tested screening compounds was carried out.
- A subset of compounds with overall highest HRs was selected.
- Analog series (ASs) were systematically extracted from these compounds to provide structural context information for activity analysis.

**Data**

- More than 6000 ASs formed by compounds with high HRs were identified.
- The ASs were organized and prioritized on the basis of different parameters.
- An open access deposition of all qualifying ASs and associated compound, assay frequency and activity information was prepared and reported herein.

**Next steps**

- The collection of ASs will be further analyzed to identify compounds and series with assay interference potential and multi-target activities.
- Open access to the data is expected to enable further progress in understanding assay interference and differentiating it from multi-target activities of small molecules.
